# Beyond BRCA1/2: Homologous Recombination Repair Genetic Profile in a Large Cohort of Apulian Ovarian Cancers

**DOI:** 10.3390/cancers14020365

**Published:** 2022-01-12

**Authors:** Antonella Turchiano, Daria Carmela Loconte, Rosalba De Nola, Francesca Arezzo, Giulia Chiarello, Antonino Pantaleo, Matteo Iacoviello, Rosanna Bagnulo, Annunziata De Luisi, Sonia Perrelli, Stefania Martino, Carlotta Ranieri, Antonella Garganese, Alessandro Stella, Cinzia Forleo, Vera Loizzi, Marco Marinaccio, Ettore Cicinelli, Gennaro Cormio, Nicoletta Resta

**Affiliations:** 1 Medical Genetics Unit, Department of Biomedical Sciences and Human Oncology, Policlinico Hospital, “Aldo Moro” University of Bari, 70124 Bari, Italy; antonella.turchiano@uniba.it (A.T.); daria.loconte@uniba.it (D.C.L.); antonino.pantaleo@uniba.it (A.P.); matteo.iacoviello@uniba.it (M.I.); rosanna.bagnulo@uniba.it (R.B.); annunziata.deluisi@uniba.it (A.D.L.); s.perrelli1@studenti.uniba.it (S.P.); s.martino5@studenti.uniba.it (S.M.); carlotta.ranieri@uniba.it (C.R.); antonella.garganese@pec.tsrm-pstrp.org (A.G.); alessandro.stella@uniba.it (A.S.); 2 Unit of Obstetrics and Gynecology, Department of Biomedical Sciences and Human Oncology, Policlinico Hospital, “Aldo Moro” University of Bari, 70124 Bari, Italy; rosalba.denola@uniba.it (R.D.N.); francescaarezzo@libero.it (F.A.); g.chiarello4@studenti.uniba.it (G.C.); ettore.cicinelli@uniba.it (E.C.); 3 Cardiology Unit, Department of Emergency and Organ Transplantation, Policlinico Hospital, “Aldo Moro” University of Bari, 70124 Bari, Italy; cinzia.forleo@uniba.it; 4 Unit of Obstetrics and Gynecology, Department of Interdisciplinary Medicine, Policlinico Hospital, “Aldo Moro” University of Bari, 70124 Bari, Italy; vera.loizzi@uniba.it (V.L.); marco.marinaccio@uniba.it (M.M.); gennaro.cormio@uniba.it (G.C.)

**Keywords:** *BRCA1/2*, HHR genes, PARPi, ovarian cancer, target resequencing

## Abstract

**Simple Summary:**

Ovarian cancer (OC) is the second most common gynecologic malignancy and the most common cause of death among women with gynecologic cancer. Despite significant improvements having been made over the past decades, OC remains one of the most challenging malignancies to treat. Targeted therapies, such as PARPi, have emerged as one of the most interesting treatments for OC, particularly in women with *BRCA1* or *BRCA2* mutations. or those with a dysfunctional homologous recombination repair pathway. The purpose of our study is to address the role of NGS-targeted resequencing in the clinical routine of OC, focusing not only on *BRCA1/2* but also on the homologous recombination repair genetic profile.

**Abstract:**

Background: Pathogenic variants in homologous recombination repair (HRR) genes other than *BRCA1/2* have been associated with a high risk of ovarian cancer (OC). In current clinical practice, genetic testing is generally limited to *BRCA1/2*. Herein, we investigated the mutational status of both *BRCA1/2* and 5 HRR genes in 69 unselected OC, evaluating the advantage of multigene panel testing in everyday clinical practice. Methods: We analyzed 69 epithelial OC samples using an NGS custom multigene panel of the 5 HRR pathways genes, beyond the genetic screening routine of *BRCA1/2* testing. Results: Overall, 19 pathogenic variants (27.5%) were detected. The majority (21.7%) of patients displayed a deleterious mutation in *BRCA1/2*, whereas 5.8% harbored a pathogenic variant in one of the HRR genes. Additionally, there were 14 (20.3%) uncertain significant variants (VUS). The assessment of germline mutational status showed that a small number of variants (five) were not detected in the corresponding blood sample. Notably, we detected one *BRIP1* and four *BRCA1/2* deleterious variants in the low-grade serous and endometrioid histology OC, respectively. Conclusion: We demonstrate that using a multigene panel beyond *BRCA1/2* improves the diagnostic yield in OC testing, and it could produce clinically relevant results.

## 1. Introduction

In 2020, it was estimated that 21,750 new cases of ovarian cancer would have been diagnosed, and 13,940 women would have died from this serious disease [1]. According to the NIH (National Cancer Institute), Ovarian Cancer (OC) represents 1.2% of all new cancer cases in the US. It is a relatively rare form, and is the fifth leading cause of all cancer deaths in females and the first among gynecological cancers. OCs are diagnosed prevalently in fertile women, mainly between 40 and 50 years of age. OC is described as the “silent killer” because few signs or symptoms allow an early diagnosis: more than 70% of OCs are diagnosed when they have already progressed to stage III or IV. According to the latest data reported by NIH, the relative survival rate after 5 years is 48.6% [2].

Epithelial Ovarian Carcinomas (EOC) is the most common type of OC. EOC is a heterogeneous group with different morphological and biological features. Currently, five different sub-categories have been identified: High-Grade Serous Carcinomas (HGSC) (70%) or Low-Grade Serous Carcinomas (LGSC) (<5%), Endometrioid (10%), Mucinous (3%), and Clear Cell (10%) [3]. Risk factors for the development of OC account for a combination of environmental and genetic components and are strictly related to hormonal levels, in particular those involved in ovulation. In fact, an increased risk has been observed in postmenopausal women using hormonal replacement therapy for longer than 10 years. The number of at-term pregnancies and the use of oral contraceptives have been shown to lower the risk [4]. Most EOCs are sporadic, while in 15–25% of cases, there is evidence of a familial or inherited component [5]. Germline mutations in *BRCA1* and *BRCA2* (high penetrance genes) account for 20–25% of all HGSC [6]. Germline mutations in any of the MisMatch repair (MMR) genes (responsible for Lynch syndrome) have been found to be less common in OC, being present in 0.5–2% of unselected OC cases [7,8]. Several reports also demonstrated the existence of other genes at medium and low penetrance (e.g., *BRIP1*, *RAD51C*, *RAD51D*, *BARD1*, and *PALB2*), which have been associated with both Breast Cancer (BC) and OC risk [7,9]. These genes encode for proteins with a fundamental role in the homologous recombination (HR) mechanism (together with *BRCA1* and *BRCA2*) by repairing DNA double-strand breaks (DSB). HR is a high-fidelity mechanism that repairs DSB using the intact sister chromatid as template, minimizing the loss of genetic information. Genes such as *BARD1*, *BRIP1*, *PALB2*, *RAD51C*, and *RAD51D* are all involved in the HR DNA repair mechanism interacting with both *BRCA1* and *BRCA2*. Briefly, BRCA1 binds the site of DSB where it coordinates the repair mechanisms downstream [10]. BARD1 and BRIP1 both directly interact with BRCA1 mediating its function [11]. PALB2 dislodge BRCA2 onto DNA breaks, thus recruiting the RAD51 complex to stabilize the single strand DNA filament [12]. RAD51C and RAD51D are two RAD51 paralogs, part of the BCDX2 complex, which regulates the RAD51 filament formation [13]. Deficiency in HR process (HRD)—due to loss of function mutation in the aforementioned genes—causes the activation of alternative repair methods (e.g., non-homologous end joining). However, these methods are rather error-prone (small DNA deletions or insertions, copy number variants, or large chromosomal rearrangements) resulting in genomic instability and triggering tumorigenesis.

Patients showing a HRD profile would benefit from therapy with PARP inhibitors (PARPi) [14]. PARPi (e.g., olaparib, niraparib, and rucaparib) act throughout a mechanism of synthetic lethality preventing the repair of single strand breaks, thus leading to cancer cells apoptosis. Olaparib is approved in the management of patients affected by advanced OC as a first-line maintenance therapy, providing a substantial progression-free survival (PFS) benefit (as demonstrated in the phase III SOLO1 clinical trial) [15,16]. Other PARPi, such as Niraparib and Rucaparib, have also been approved in the United States and Europe as maintenance therapy regardless the HRD status, mostly in patients with platinum-sensitive recurrent OC [17,18,19].

Here, we report a molecular characterization of a cohort of 69 patients from Apulia (in the south-east of Italy) with primary EOC. In this study, 69 EOC samples were analyzed by next-generation sequencing (NGS) using a multigene panel including five HR pathways genes (*BARD1*, *BRIP1*, *PALB2*, *RAD51C*, and *RAD51D*), which represent the most frequently mutated genes (besides *BRCA1/2*) in OC [20].

The tumor samples were tested using an Oncomine™ BRCA Research Assay Thermo Fisher, Waltham, MA, USA, Devyser’s CE-IVD, and an Ion AmpliSeq Custom Panel (Thermo Fisher, Waltham, MA, USA) designed with five genes related to HR repair (*BARD1*, *BRIP1*, *RAD51D*, *RAD51C*, and *PALB2*). This study aimed to (1) carry out a clinical-molecular screening of 69 patients, to verify the correlation between type of mutation and clinical status of patients; (2) to define the mutation profiles of HR genes; and (3) evaluate the usefulness of multigene panel in terms of prevention protocols and therapeutic approaches.

## 2. Results

### 2.1. Cohort Characteristics

All the clinical and demographic characteristics of our cohort have been summarized in Table 1. The median age of OC onset was 61 years (range from 31 to 88). As far as concerns the histology, most cases were classified as serous (45 out of 69), with a prevalence of high-grade serous type (38 out of 45). Among the other 24 OC cases several histological types have been identified, including endometrioid (10), clear cell (5), and mucinous (1).

### 2.2. Overall Mutation Spectrum

The somatic testing of our samples showed the presence of 19 pathogenic variants (27.5%). The distribution of mutations revealed that most (21.7%) of the 69 patients displayed a deleterious mutation in either *BRCA1* or *BRCA2*, whereas 5.8% harbored a pathogenic variant in one of the HR pathway genes. 

**Figure 1:**
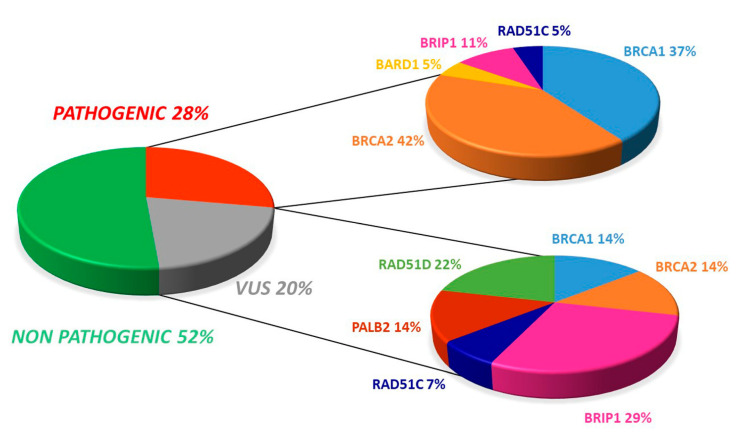
Overall distribution of deleterious and uncertain significance variants in our cohort.

Additionally, there were 14 (20.3%) uncertain significant variants (VUS), with four occurring in *BRCA1/2* while ten involved at least one HR gene (Figure 1). 

**Figure 2:**
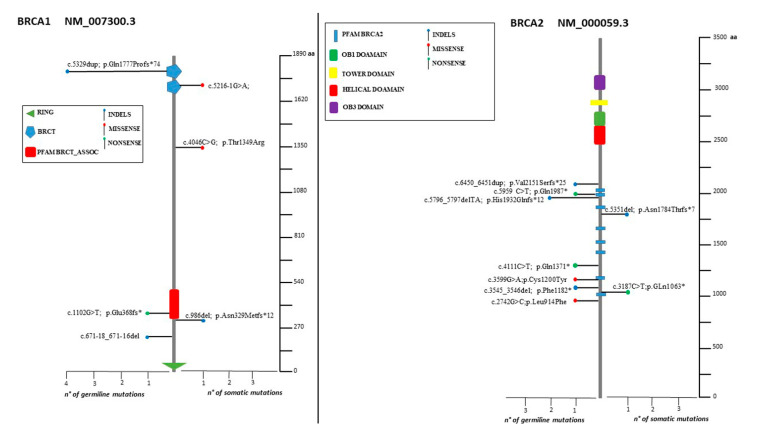
Lolliplots showing germline and somatic variant spectrum (both pathogenic and VUS) throughout the whole protein sequences of *BRCA1* and *BRCA2*. The vertical black scale bar represents the length (amino acids) of the protein sequence. The horizontal bar represents the number of patients affected by the specific mutation. Each lolliplot represents a variant identified in this study. The blue lolliplot identifies a shorts indels mutation (deletion or duplication). The red lolliplot indicate a missense variant, while the green ones a nonsense mutation.

**Figure 3:**
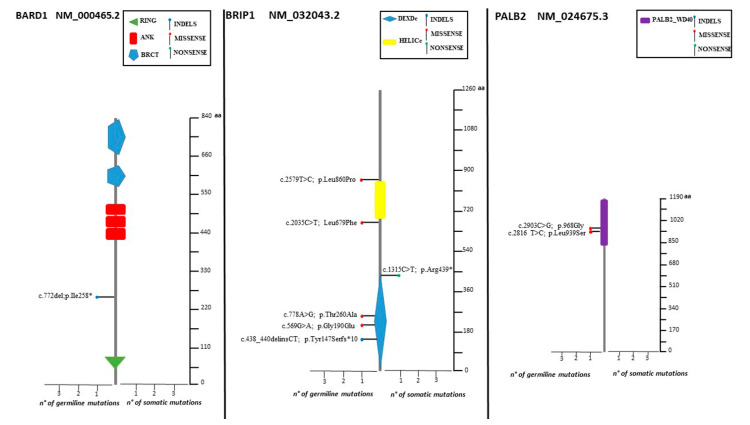
Lolliplots showing germline and somatic variant spectrum (both pathogenic and VUS) throughout the whole protein sequences of *BARD1*, *BRIP1*, and *PALB2*. The vertical black scale bar represents the length (amino acids) of the protein sequence. The horizontal bar represents the number of patients affected by the specific mutation. Each lolliplot represents a variant identified in this study. The blue lolliplot identifies a shorts indels mutation (deletion or duplication). The red lolliplot indicate a missense variant, while the green ones a nonsense mutation.

**Figure 4:**
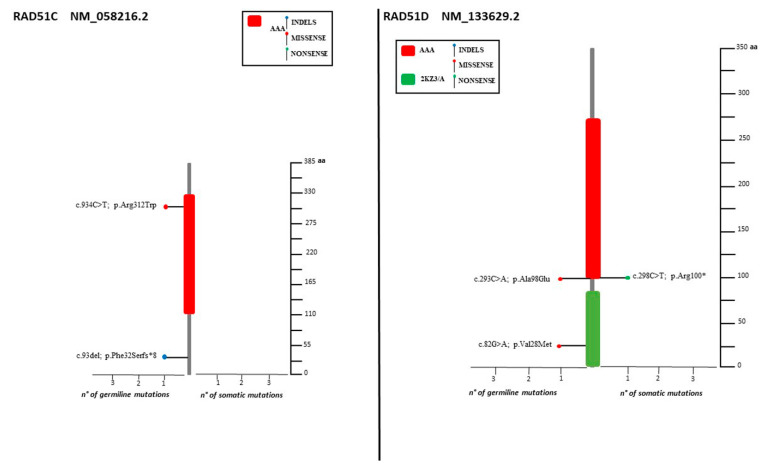
Lolliplots showing germline and somatic variant spectrum (both pathogenic and VUS) throughout the whole protein sequences of *RAD51C* and *RAD51D*. The vertical black scale bar represents the length (amino acids) of the protein sequence. The horizontal bar represents the number of patients affected by the specific mutation. Each lolliplot represents a variant identified in this study. The blue lolliplot identifies a shorts indels mutation (deletion or duplication). The red lolliplot indicate a missense variant, while the green ones a nonsense mutation.

The spectrum of identified mutations includes 19 single-nucleotide variants (6 nonsense, 12 missense, and 1 splice variant) and 14 indels (8 deletions, 5 duplications, and 1 indel) (Figure 2, Figure 3 and Figure 4).

**Figure 5:**
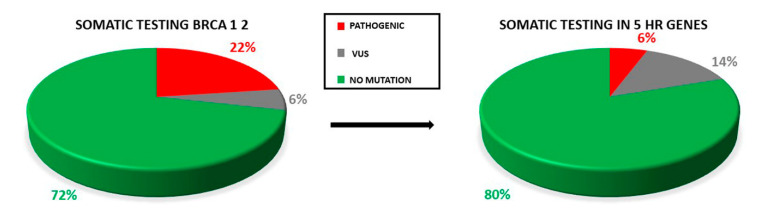
Proportion of pathogenic variants and VUS in testing only BRCA1/2 genes and retesting all the OC samples for the five HR genes. The rate of VUS detection is increased.

Only two samples displayed the co-occurrence of two variants: two VUS (*RAD51D*: c.82G>A; *RAD51D*: c.293C>A) in one case, one VUS and one pathogenic variant (*BRCA1*: 671-18_671-16del, *BARD1*: c.772delA) in the second case. Retesting all the samples for the HR genes panel increases the yield of VUS detected (Figure 5).

### 2.3. Distribution of Mutation in BRCA1/2

Of the 19 pathogenic variants, seven (36.8%) occurred in *BRCA1* and eight involved *BRCA2* (42.1%). Regarding VUS, both *BRCA1* and *BRCA2* displayed two variants (14.3%, Figure 1 and Figure 2).

Two deleterious mutations were recurrent (*BRCA2*: c.5796_5797del, *BRCA1*: c.5329dup) in two and four individuals, respectively (Figure 2). 

### 2.4. Distribution of Mutation in Non BRCA Genes

Four subjects were identified as having deleterious variants in one of the HR pathway genes *(BARD1*: c.772del, *RAD51C*: c.93del, *BRIP1*: c.438_440delinsCT, *BRIP1*: c.1315C>T) (Figure 3 and Figure 4).

The VUS distribution among the five HR genes was as follow: *BRIP1* (28,5%), *RAD51D* (21.5%), *PALB2* (14.3%), and *RAD51C* (7.1%) (Figure 1, Figure 3 and Figure 4). No recurrent variant was detected.

### 2.5. Germline Mutation Status

**Figure 6:**
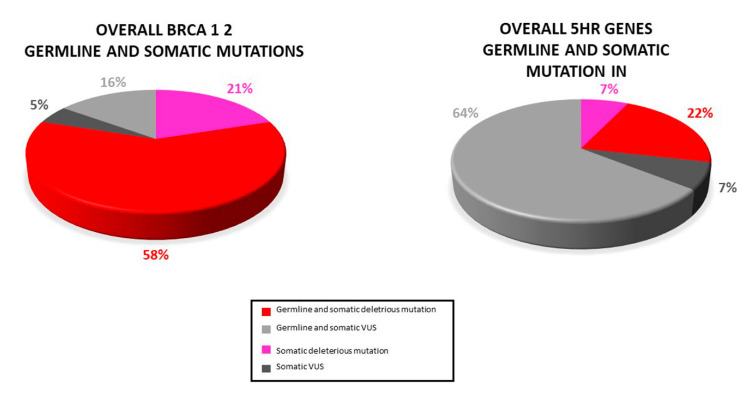
Distribution of somatic and germline variant detected in our study: Most somatic variants (pathogenic and VUS) were also found in the paired blood sample.

Based on PB samples availability, the germline occurrence of variants was evaluated. A total of 24 (34.8%) individuals presented both germline and somatic mutations: 11 (15.9%) deleterious *BRCA1/2* mutations, 3 (4.3%) HR genes pathogenic mutation, 3 (4.3%) *BRCA1/2* VUS, and 9 (13%) HR genes VUS (Figure 6).

Our analysis revealed that the majority (78.8%) of the somatic variants detected was also found in PB. However, three deleterious mutations (*BRCA1*: c.986del, *BRCA2*: C.3187C>T, *BRCA2*: c.5351del) as well as two VUS (*BRCA1*: c.4046 C>G, *RAD51D*: c.298C>T) were not present in PB. Because of the lack of the corresponding PB sample, for two somatic variants (*BRCA1*: c.5216-1G>A, *BRIP1*: c.1315C>T) the germline testing was not possible. 

### 2.6. Mutations and Histology

**Figure 7:**
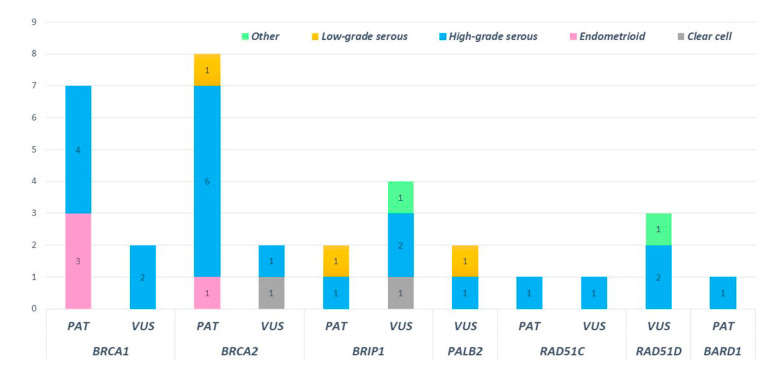
Distribution of pathogenic and uncertain significance variants according to the ovarian cancer histology of our cohort.

High-grade serous histotypes displayed a heterogeneous spectrum of variants with a prevalence of *BRCA1/2* mutations. Eighteen high-grade serous tumors out of thirty-eight (47.4%) were not affected by mutation in any tested genes. Three out of four deleterious mutations in HR genes were found in subjects with high-grade serous histology. Of note, a pathogenic mutation in *BRIP1* was detected in low-grade serous histology (*BRIP1*: c.438_440delinsCT). Moreover, ten deleterious variants in *BRCA1* and *BRCA2* were detected in patients showing the high-grade serous histotype. Among non-serous histology tumors, four endometrioid cancers were mutated, revealing three deleterious mutations in *BRCA1* and one in *BRCA2* (Figure 7).

### 2.7. BRCA1 and BRCA2 Copy Number Variant

In 67 patients of our cohort, for whom PB sample was available, MLPA analysis of *BRCA1/2* was carried out. None revealed a CNV alteration. 

## 3. Discussion

Several pieces of evidence have emerged regarding variations in HR genes other than *BRCA1/2* (e.g., *BRIP1*, *RAD51C*, and *RAD51D*) associated with a high risk of OC [21,22]. These new findings might lead to the implementation of novel therapeutic procedures, such as PARPi. In this scenario, NGS is rapidly developing as a powerful tool that allows the analysis of multiple genes simultaneously and providing both prognostic and predictive value for OC. The importance of genetic testing in clinical practice is constantly increasing; although, it is generally limited to the *BRCA1/2* screening. Herein, we investigated the mutational status of both *BRCA1/2* and 5 HR genes (*BRIP1*, *RAD51C*, *RAD51D*, *PALB2*, and *BARD1*) in 69 unselected OC, with the aim of evaluating the advantages of multigene panel testing in the everyday clinical practice.

Overall, our pathogenic variants’ frequency of 21.7% in *BRCA1/2* (10.1% and 11.6% for *BRCA1* and *BRCA2*, respectively) is within the range of other published studies on OC, ranging from 3% to 15% [21,23,24,25]. 

As far as concerns non-*BRCA* genes, we detected a deleterious mutation in 5.8% of our cohort patients (2.9% in *BRIP1*, 1.5% each in *BARD1* and *RAD51C*), which is widely consistent with previous literature [21,23,24]. Pathogenic variants in *BRIP1*, *BARD1*, and *RAD51C* are all acknowledged as being associated with an increased risk of OC [22,26,27].

Hence, identifying deleterious variants in these latter genes might have a clinical impact resulting in a change in the patients’ management (in terms of either therapeutic approach or testing relatives) [28,29,30]. In *BRCA1* and *BRCA2*, multiple mutation cluster regions have been observed and are associated with relatively different BC and OC risks [31]. In our cohort, five out of seven *BRCA1* pathogenic mutations are located from c.5216 to c.5563, which has been described as a region associated with higher BC risk [10]. Deleterious mutation of *BRCA2* were detected in the coding sequence region comprised between c.3187 and c.6450, which is consistent with the higher OC risk compared to BC risk associated to mutations in this cluster [10]. To our knowledge, no significance differences have been reported so far in the assessment of OC risk vs. BC risk in correlation with the distribution of the pathogenic variants along *BRIP1*, *RAD51C*, and *BARD1* coding sequences. Nevertheless, truncating variants in *BARD1*, *BRIP1*, and *RAD51C* are associated with a moderate risk of female BC, generally accounting for less than 1% of Breast Cancers [32].

The National Comprehensive Cancer Network (NCCN) guidelines report on mutations in *BRIP1* and *RAD51C* as being a cause for potential increase in breast cancer risk (specifically for triple negative breast cancers), but there is insufficient evidence for risk management [33].

Two recurrent variants were detected (*BRCA1*: c.5329dup and *BRCA2*: c.5796_5797del). Both have already been reported as recurrently found in a large cohort of individuals, particularly in Italian cohorts [34,35,36,37,38].

Of note, the multigene panel approach provides a higher yield of VUS in non *BRCA* genes (5.8% of VUS in *BRCA1/2* compared to 14.5% in HR genes). This result was not unexpected since the rate of the “hard to interpret” variants increase with the introduction of a multigene panel [39]. Consequently, a prioritization strategy is useful to select variants with the highest probability of being associated with OC risk. 

The assessment of germline mutational status showed that a minority of variants (three pathogenic mutations in *BRCA1/2* and two VUS in *BRCA1* and *RAD51D*) were not detected in the corresponding blood sample. Therefore, most of our mutated cases revealed a hereditary predisposition to OC. To date [40,41], 13–18% of OC is associated with germline mutations in *BRCA1*/2, whereas an additional 4% with inherited mutations in other susceptibility genes [41], which are both in concordance with our overall germline deleterious mutation frequency (17.4% for *BRCA1/2* and 4.3% for HR genes). As a matter of fact, simultaneous germline and somatic analysis are needed in clinical practice to guide the OC patients’ care. 

Concerning the distribution of variants among the different histotypes, 10 high-grade serous subtypes (26.3%) out of 38 individuals revealed a *BRCA1/2* pathogenic mutation, and three carried a pathogenic mutation in one of the HR genes. These results further confirm previous literature reports [6,42,43,44]. Remarkably, four (40%) endometrioid cancers out of ten in our cohort were found to harbor three deleterious mutations in *BRCA1* and 1 in *BRCA2*. This finding differs from other published research, which refer to a lower *BRCA1/2* mutation rate in the endometrioid histology [6,43]. Of note, in two patients affected by low serous OC, two pathogenic mutations in *BRIP1* (c.438_440delinsCT) and in *BRCA2* (c.6450dup), respectively, were disclosed. Generally, activating somatic mutations in mitogen-activated protein kinase pathway genes, most commonly in *KRAS*, *BRAF*, and *NRAS*, are common in LGSC. Such genetic alterations promote tumorigenesis through constitutive activation of MAPK/ERK pathway. Considering this evidence, therapies based on MEK and *BRAF* inhibitors use have shown efficacy and improved clinical outcomes [45]. Deleterious germline mutations in *BRIP1* have been rarely reported as being associated with low-grade serous histology [43,46]. Weber-Lassalle et al. [47] have previously reported the occurrence of BRIP1 deleterious variants in tumors with low-grade histology. Similarly, *BRCA2* pathogenic mutation are unusual in LGCS. Previous work reported *BRCA1/2* deleterious mutations as very rare in the low-grade histology [48,49]. Having identified pathogenic mutations in genes such as *BRCA2* and *BRIP1*, albeit in a small percentage of cases, the possibility for these patients to access therapies that were previously precluded opens up. Furthermore, the *BRCA2* mutated-LGCS patient presented also a somatic KRAS mutation (G12A). This result further confirms that dysregulation of the MAPK/ERK pathway is important in tumors with low-grade histology.

The majority (55%) of individuals in our cohort had neither pathogenic mutations nor deletions or duplications. We speculate that other mechanisms could be possibly involved in the OC onset in our cohort. For instance, a large body of research points out to the role of *BRCA* methylation in causing *BRCA* dysfunction in OC [50]. Thus, future studies should include the assessment of BRCA methylation status to evaluate the reliability of this new molecular entity in the clinical management. Similarly, somatic and germline CNV evaluation for genes other than *BRCA1/2* should be performed to ascertain frequency and involvement of large duplications/deletions in OC, demanding for the implementation of a more sophisticated NGS system and bioinformatic pipeline.

Furthermore, mutations in MMR genes should also be investigated, specifically in tumors with clear cell and endometrioid histology. 

Clinically, the relatively short follow-up times in our study, lower than the average survival of women affected by EOC (5 years), did not achieve a statistically significant result concerning the PFS in patients with mutations versus patients without mutations. However, the concurrent diagnosis of high HRD and EOC is associated with an increased PFS. Therefore, the HRD score can be translated as a valid prognostic score particularly useful in a precision medicine scenario [19,51]. The synthetic lethality provoked by PARPi is based on HRD (due to *BRCA* mutations and the BRCAness phenotype), even if the clinical response to PARPi depends on time and chemosensitivity. This variability can be explained throughout PARPi resistance due to tumoral restoration of HRR functions, thus evading the combination of the genetical and iatrogenic “two hits” (HRD-PARPi). In other words, the HRD status is dynamic, and therefore is crucial the early introduction of PARPi in the EOC treatment timeline. In fact, in the SOLO-1 trial, more than 50% of *BRCA 1/2* mutated women with advanced EOC who gained a complete response after frontline chemotherapy, experienced at least 5 years free from EOC’s relapses. Interestingly, clinical response rates and survival outcomes of the PFS are similar in cases of somatic or germline mutation of *BRCA 1/2*. Notably, in case of platinum sensitivity, we can predict a positive response to PARPi. However, considering all the HRD panels, there is an evident trend of higher PFS in crescent order: HRD-negative, HRD-positive, and *BRCA1/2*-mutant subgroups [52]. 

## 4. Materials and Methods

### 4.1. Patient Recruitment

Sixty-nine patients with a primary diagnosis of malignant epithelial OC were referred to the UOC of Obstetrics and Gynecology at the Università Degli Studi di Bari, from September 2018 to October 2020. Neither age at diagnosis nor tumor histotype was used as selection criteria. Clinicopathological characteristics of the patients were collected to analyze the distribution of identified mutations in subgroups of patients. Fresh cancer tissues were collected from all 69 patients. Peripheral blood (PB) samples were withdrawn (when available) to eventually assess both the presence of a corresponding germline mutation after mutation identification in the tumor sample and to verify the presence of germline copy number variation (CNV) in *BRCA1/2* genes in patients who tested negative for somatic analysis for *BRCA 1/2* genes. In case of a positive genetic test for a germline mutation, post-test genetic counseling was offered to all patients to discuss the risk for their relatives. Written informed consent was obtained from patients to perform the genetic tests for diagnostics and research purposes on peripheral blood and tissue biopsy according to the local ethic committee’s policy (approval code study: 5517 N0017671|26/02/2018, Policlinico of Bari, Italy).

### 4.2. Next-Generation Sequencing

Genomic DNA was isolated from PB and biopsy samples using the QIAamp Mini Kit (Qiagen, Hilden, Germany), according to the manufacturer’s instructions, and quantified on a BioSpectrometer Plus (Eppendorf, Hamburg, Germany) and Qubit ds DNA HS Assay Kit on Qubit 2.0 Fluorimeter (Invitrogen, Carlsbad, CA, USA) following the manufacturer’s instructions. The *BRCA1/2* analysis was performed using Oncomine™ BRCA Research Assay (ThermoFisher, Carlsbad, CA, USA) for 60 individuals. 

An Ion AmpliSeq Custom Panel was designed online using Ion AmpliSeq Designer v.7.06 (http://www.ampliseq.com/ accessed on 27 March 2019) (Thermo Fisher, Waltham, MA, USA) to analyze the CDSs (+/− 25 bp of intronic flanking regions) of five HR genes (*BRIP1, RAD51C, RAD51D, BARD1*, and *PALB2*). The final custom panel was composed of 197 amplicons divided into three primer pools for a total of 19.01 kb of target size DNA and coverage of 98.78%. Library preparation was performed using the Ion AmpliSeq^TM^ Library Kit Plus (Life Technologies, Carlsbad, CA, USA) following the manufacturer’s instructions. The equimolar concentration of each library was used to prepare templates for clonal amplification. Emulsion PCR was carried out on Ion OneTouch 2 Instrument (Thermo Fisher Scientific, Carlsbad, CA, USA) using the Ion PGM^TM^ Hi-Q View OT2 Kit (Thermo Fisher Scientific, Carlsbad, CA, USA). Templates were enriched using Ion OneTouch ES (Enrichment System) (Thermo Fisher Scientific, Carlsbad, CA, USA), loaded on a 316v2/318v2 chip, and sequenced on an Ion Torrent Personal Genome Machine (Life Technologies, Carlsbad, CA, USA) using Ion PGM Hi-Q View Sequencing Kit (Thermo Fisher Scientific, Carlsbad, CA, USA). Nine samples were tested for BRCA1/2 by the Devyser’s CE-IVD library prep kit (Devyser, Hägersten, Sweden). Sequencing run was performed on an Illumina Miseq instrument (Illumina, San Diego, CA, USA) following the manufacturer’s instructions using a standard flow cell (Illumina, San Diego, CA, USA) and V2 300 cycle cartridge (Illumina, San Diego, CA, USA). To validate variants detected with NGS and to confirm the corresponding presence in PB samples, direct sequencing was performed using the BigDye Terminator v1.1 Cycle Sequencing Kit (Applied Biosystems, Waltham, MA, USA) on SeqStudio Genetic Analyzer (Applied Biosystems, Waltham, MA, USA) following the manufacturer’s instructions. 

### 4.3. Variant Analysis and Data Interpretation

For the Oncomine *BRCA* panel, PGM sequencing produced mean reads per patient of about 600,000 with the Mean Read Length of 108 bp. The average base coverage depth was about 3626×. The mean percentage of regions of interest (ROI), covered at least by 100×, was 100.00% with a Uniformity of base coverage of 99.98%. For the custom panel including 5 HR genes, mean reads per patient are about 550,000 with a mean read length of 115 bp. The average base coverage depth was about 3000×. The mean percentage of regions of interest (ROI), covered at least by 100×, was 98.71%, with uniformity of base coverage of 98.48%. Data analysis was performed using Torrent Suite version 5.0.4 and Ion Reporter version 5.14.1.2 (Thermo Fisher Scientific, Carlsbad, CA, USA). Alamut Visual 2.13-0 (Sophia Genetics, Lausanne, Switzerland) was used to visually check the sequencing data and the corresponding BAM file. For the remaining nine samples tested by the Devyser’s CE-IVD kit for *BRCA1/2*, a mean of 2,245,135 reads with a mean read length of 147 bp per sample was obtained. The average coverage depth was about 11,019, with uniformity of base coverage of 100%. Data analysis was performed by MiSeq™ Control Software 3.1 (Illumina, San Diego, CA, USA). FASTQ files were analyzed with Amplicon Suite software version 3.1 (Illumina, San Diego, CA, USA) according to the manufacturer’s instructions. Variants were analyzed Online databases, including dbSNP (database the single nucleotide polymorphism database), 1000 Genomes, ClinVar, EXAC (exome aggregation consortium), COSMIC (catalog of somatic mutations in cancer), ESP (exome sequencing project), and ENIGMA (Evidence-based Network for the Interpretation of Germline Mutant Alleles, for *BRCA 1 2* variant annotation). Pathogenicity prediction programs (e.g., PolyPhen2, SIFT, Mutation Taster) and splice prediction programs were used to check out variants not formerly described.

### 4.4. MLPA

To assess the presence of germline Copy Number Variation (CNV) in BRCA1/2 genes, MLPA (Multiplex ligation-dependent probe amplification MRC Holland^®^ (Amsterdam, The Netherlands)), *BRCA1* P002 D1, and *BRCA2* P045 D1 were performed on blood samples (when available) of patients who tested negative for somatic analysis for BRCA1/2 genes. Data analyses were performed with Coffalyser.Net-MRC Holland software^®^ (Amsterdam, The Netherlands). 

### 4.5. KRAS and BRAF Mutational Analysis

Mutational analysis of the *BRAF* and *KRAS* genes was performed on OC samples by real-time polymerase chain reaction (Therascreen, Qiagen, Hilden, Germany). Tumor specimens were screened for hot-spot mutations in codon 600 of the *BRAF* gene and in codons 12 and 13 of the *KRAS* gene.

## 5. Conclusions

Using a multigene panel testing in patients affected by OC, rather than testing *BRCA1/2* alone, could generate clinically relevant results. Further research is warranted to verify the clinical implications to develop a standard clinical multigene panel and narrow down the number of candidates VUS detected. 

## Figures and Tables

**Table 1:** Clinical and demographic characteristics of our cohort.

Clinical Characteristic	
Age of Diagnosis	# Patients
≤40	2
40–50	13
50–60	19
60–70	16
>70	19
Tumor Histology	
High-grade serous	38
Low-grade serous	7
Clear cell	5
Endometrioid	10
Mucinous	1
others	8

## Data Availability

The data presented in this study are available on request from the corresponding author.
